# Violence and threat exposure is associated with frontostriatal alterations during risky decision-making in children with co-morbid ADHD and disruptive behavior disorders

**DOI:** 10.3389/fpsyt.2026.1799471

**Published:** 2026-05-08

**Authors:** Joseph Aloi, Olivia K. Murray, Kathleen I. Crum, Matthew C. Aalsma, Mario Dzemidzic, Leslie A. Hulvershorn

**Affiliations:** 1 Department of Psychiatry, Indiana University School of Medicine, Indianapolis, IN, United States; 2 Adolescent Behavioral Health Research Program, Indiana University School of Medicine, Indianapolis, IN, United States; 3 Medical Scientist Training Program, Indiana University School of Medicine, Indianapolis, IN, United States; 4 Department of Pediatrics, Indiana University School of Medicine, Indianapolis, IN, United States; 5 Department of Neurology, Indiana University School of Medicine, Indianapolis, IN, United States; 6 Department of Radiology and Imaging Sciences, Indiana University School of Medicine, Indianapolis, IN, United States

**Keywords:** disruptive behavior disorder, early life adversity (ELA), fMRI, neuroimaging, risky substance use

## Abstract

**Introduction:**

Early life adversity (ELA) is prevalent in the US and is associated with a number of risky behaviors, including substance use. Recent work has shown that specific forms of ELA, including violence exposure and threat exposure, is associated with alterations in reward and attentional neuro-circuitries implicated in risky decision-making. Although a couple of studies have examined the relationship between ELA and neural alterations in youths at high risk for substance use disorders (SUDs), none of these studies have examined neural functioning during risky decision-making.

**Methods:**

One-hundred twenty-three participants aged 11–12 recruited within a US community completed the Balloon Analogue Risk Task (BART) during functional magnetic resonance imaging (fMRI). We specifically defined ELA as exposure to violence and trauma (VTE), which was measured using the Screen for Adolescent Violence Exposure (SAVE) and the UCLA PTSD reaction index. Additionally, substance use outcomes, such as problematic substance use, were collected every 6 months following fMRI. All data preprocessing, individual-level analyses, and group analyses were performed using Analysis of Functional NeuroImages.

**Results:**

Individuals with high levels of VTE showed reduced BOLD response when making safe choices within the anterior insula, inferior frontal gyrus, and caudate nucleus. VTE was also associated with problematic substance use at follow-up. Logistic regression analyses showed that BOLD responses when making risky choices in these regions also predicted problematic substance use at follow-up.

**Discussion:**

The current findings provide evidence of neural alteration during risky decision-making in children with greater VTE, specifically in neural responses differentiating between safer and riskier options. These findings may suggest that children with VTE have alterations in generating neural signals that help differentiate between safer and riskier options, resulting in impairments in goal-directed behaviors and potentially predisposing them to risky substance use in adolescence.

## Introduction

One in eight children in the US experience significant early life adversity (ELA) by the age of eighteen (1). ELA is defined as exposure to chronic or severe stressful life events during childhood or adolescence, which exceeds the child’s coping resources (2). ELA can take several forms, including, but not limited to violence exposure and threat exposure (2). Additionally, individuals can have heterogeneous neurobiological and psychological responses to ELA, including violence exposure and threat response (3, 4). Some individuals may experience a normative stress response where they return to a baseline level of functioning, while others may experience a traumatic stress response including post-traumatic stress disorder. However, a number of individuals may experience pathogenic stress, where they do not develop a directly trauma-related disorder but the stress response puts them at greater risk for psychiatric disorders, including substance use disorders (3). It is also possible that the biological impact of adversity or stressors may be context-dependent (4). Research indicates that childhood violence and threat exposure is associated with increased risk for developing substance use disorders (SUDs) during adolescence and adulthood (5, 6) through increased engagement in behaviors that increase risk to health (7). This phenomenon is believed to stem from the adverse effects of violence and threat exposure on neurodevelopment of systems underlying reward and executive functioning (7).

Violence and threat exposure has been linked to effects on the reward and executive function systems, which are involved in risky decision-making (8). Animal research has demonstrated that ELA exposure disrupts the development of frontostriatal circuitry critical for reward processing and learning (9). Functional MRI (fMRI) studies in humans have also indicated that violence and threat exposure is linked to reduced neural responsiveness to reward within the striatum and prefrontal cortex (10–15). Additionally, violence and threat exposure has been associated with impairments in the neuro-circuitries underlying executive function (11, 16), which play a role in risky decision-making (17). Previous studies have found that children who have experienced maltreatment exhibit reduced activity within rostromedial prefrontal and parietal cortices during incongruent trials during the Affective Stroop task (11). During a go/no-go task, ELA defined broadly was associated with reduced activity within lateral frontal cortex when making errors (18). Theoretical work has suggested that ELA is associated with reduced reward-related activity in frontostriatal networks that facilitate learning and decision-making (19). In summary, childhood violence and threat exposure is associated with impairments in frontostriatal and frontoparietal networks, both of which are involved in the neural processes of risky decision-making.

Furthermore, ELA, including violence and threat exposure, is associated with adverse outcomes associated with impulsive and risky decision-making, such as initiation of substance use (20). One longitudinal study showed that ELA (defined broadly) in early childhood was associated with impaired inhibitory control during middle childhood and increased substance use onset by age 14 (21). Moreover, childhood trauma is associated with disrupted connectivity between salience network and lateral frontal regions, and this disruption predicts reduced executive functioning and high-risk drinking behaviors in adolescence (22). Theoretical models of adolescence suggest that these networks are undergoing considerable neurodevelopment during adolescence (23), suggesting that alterations in development of these networks may contribute to adverse psychiatric outcomes related to violence and threat exposure, such as substance use.

However, many studies to date have examined violence and threat exposure in the context of populations with internalizing disorders (14, 24–27) or do not report co-morbid psychopathology (18). Although violence and threat exposure is associated with externalizing disorders (28, 29), only a couple of studies have examined violence and threat exposure within children or adolescents with externalizing disorders (10, 11, 15, 30). However, many of the studies examining the effect of ELA in populations with externalizing symptoms have focused either on neural activity during emotion regulation (11, 30) or reinforcement learning (10, 15). No studies to date have examined the impact of violence and threat exposure on neural activity during risky decision-making in youths with externalizing disorders.

Although violence and threat exposure is associated with the development of substance use and substance use disorders in adolescence (5, 6), only a few studies to date directly examine the neurobiology that may underly this increased risk. One such study showed that altered connectivity between the dorsal anterior cingulate cortex, anterior insula, and other regions in executive control networks mediated the relationship between childhood trauma and alterations in executive functioning. This study further showed that alterations in executive functioning predicted risky drinking behaviors (22). Another study showed that adverse childhood experiences were associated with reduced connectivity within default mode, salience, and frontoparietal networks and that reduced connectivity within default mode and salience networks was associated with drinking behaviors (31). However, these studies used resting-state fMRI data, and so the role of neural activity specifically associated with risky decision-making could not be assessed.

The current study aimed to investigate the impact of violence and threat exposure on neural activity during risky decision-making in children with externalizing disorders and predict problematic substance use. Children with externalizing disorders, defined in this study as co-morbid ADHD and disruptive behavior disorders, and low and high levels of violence and threat exposure performed a task where they had to make risky versus safe decisions while undergoing fMRI. Based on prior empirical (10) and theoretical work (19, 32), we hypothesized that violence and threat exposure would be associated with reduced activity within lateral frontal and striatal networks when making risky decisions and that reduced activity within these networks would predict substance use initiation.

## Methods

### Sample

Two-hundred sixty-seven English-speaking, right-handed, 11–12 year-old participants were recruited in a US community-based sample as part of an ongoing longitudinal study aimed at investigating the relationship between family history of SUD, risky decision-making, and substance use in youths with disruptive behavior disorders (33–38). After consent and assent, interviews were conducted either in-person at study facilities or via Zoom Health (due to COVID restrictions) by child mental health clinicians using a DSM-5 modified version of the K-SADS-PL (39) to determine eligibility. All participants in the current study met DSM-5 criteria for diagnosis of ADHD *and* a disruptive behavior disorder (i.e., oppositional defiant disorder, conduct disorder, or DBD other specified). See **Supplementary Material** for exclusion criteria.

### Ethical considerations

This study was approved by the Indiana University Institutional Review Board. Written consent was obtained from all parents, and written assent was obtained from all children participating in the current study.

### Groups

#### Early life adversity

To identify children with low versus high levels of violence and threat exposure (VTE), we used a latent variable approach in SPSS 28.0 on all 267 children in the sample. We conducted a factor analysis with maximum likelihood factoring on the following measures of stress and violence exposure: total count of nine items from Part 2 of the UCLA PTSD reaction index (see supplement for the items that were used) (40) and scores on the three subscales of the Screen for Adolescent Violence Exposure (Indirect Violence, Traumatic Violence, and Physical/Verbal Abuse) (41). Since we were interested in extracting an overall VTE variable, we extracted one factor from this factor analysis. Scores on the resulting VTE latent variable were calculated using the regression method. We then split the sample into low and high VTE groups based on a mean split of the resulting z-scores (low VTE if z-score<0, high VTE if z-score>0). For further details regarding our latent variable procedures, see **Supplementary Material**.

Of the 267 children enrolled in the parent study, 58 children were excluded from the current study as they had completed only the questionnaire portions but not fMRI. Forty-nine children did not meet criteria for a DBD diagnosis and were therefore excluded from the current study. Of the 160 remaining children, 37 were excluded due to data quality issues (motion or other imaging artifact, taking medication on the morning of the study), resulting in the final sample of N = 123 participants. This sample included n=69 children with low VTE (39 without a family history of SUD and 30 with a family history of SUD) and n=54 children with high VTE (24 without a family history of SUD and 30 with a family history of SUD) as detailed in **Table 1**.

**Table 1:** Demographic information and clinical variables.

	Lo VTE/FH-	Hi VTE/FH-	Lo VTE/FH+	Hi VTE/FH+	F/chi-square	Comparison
N	39	24	30	30		
Age (SD)	11.9 (0.51)	11.9 (0.61)	11.9 (0.55)	12.0 (0.53)	0.45 1.01 0.69	
% Male	69.2%	70.8%	50.0%	76.7%	5.40	
Race	17.9% AA 71.8% Cauc 10.3% Mixed	33.3% AA 50.0% Cauc 16.7% Mixed	40.0% AA 56.7% Cauc 3.3% Mixed	20.0% AA 63.3% Cauc 16.7% Mixed	8.37	
Ethnicity (% Hispanic)	7.7%	8.3%	10.0%	13.3%	0.68	
Verbal IQ (SD)	108.5 (14.15)	109.5 (16.17)	107.4 (15.89)	104.4 (14.46)	1.29 0.13 0.53	
Performance IQ (SD)	105.6 (15.65)	105.0 (16.29)	103.8 (14.71)	101.5 (15.30)	0.70 0.23 0.07	
Tanner Stage (SD)	2.1 (1.20)	2.5 (1.26)	2.3 (1.48)	2.3 (1.07)	0.00 0.68 0.62	
Parental Education (SD)^a^	2.9 (1.22)	2.8 (1.20)	2.7 (1.39)	2.1 (0.96)	5.19* 2.51 1.24	FH->FH+
SAVE IV (SE)	7.7 (0.19)	23.6 (1.65)	9.2 (1.48)	23.3 (1.48)	0.17 103.06* 0.34	Hi VTE>Lo VTE*
SAVE TV (SE)	0.4 (0.71)	2.5 (0.90)	0.4 (0.81)	5.3 (0.81)	2.91 19.25* 2.88	Hi VTE>Lo VTE*
SAVE PA (SE)	0.9 (0.40)	3.9 (0.51)	1.0 (0.45)	5.3 (0.45)	2.95 64.60* 2.22	Hi VTE>Lo VTE*
UCLA (SE)	0.3 (0.17)	1.0 (0.22)	0.4 (0.19)	2.2 (0.19)	11.33* 39.13* 8.34*	FH+>FH-* Hi VTE>Lo VTE* Hi VTE/FH+>Hi VTE/FH->Lo VTE/FH-=Lo VTE/FH+*
UPPS-LPL (SE)	2.2 (0.09)	2.2 (0.11)	1.97 (0.10)	2.25 (0.10)	0.97 2.09 2.09	
UPPS-LPS (SE)	2.1 (0.07)	1.8 (0.09)	2.0 (0.08)	2.1 (0.08)	1.33 1.11 3.44	
UPPS-NUR (SE)	2.5 (0.10)	2.7 (0.13)	2.2 (0.12)	2.7 (0.12)	1.39 10.56* 2.07	Hi VTE>Lo VTE*
UPPS-PUR (SE)	2.5 (0.10)	2.8 (0.14)	2.3 (0.12)	2.6 (0.12)	3.16 6.80* 0.08	Hi VTE>Lo VTE*
UPPS-SS (SE)	2.8 (0.10)	2.9 (0.13)	2.6 (0.11)	3.0 (0.11)	0.39 4.82* 1.08	Hi VTE>Lo VTE*
% CD	7.7%	4.2%	10.0%	10.0%	0.80	
% ODD	64.1%	70.8%	76.7%	76.7%	1.84	
% Other DBD	17.9%	12.5%	20.0%	13.3%	0.83	
% Trauma-Related Disorder (Past)	5.1	8.3%	16.7%	13.3%	2.77	
% Anxiety-Related Disorder (Past)	0%	0%	0%	0%	n/a	

*N=*123, *indicates significant differences at *p* <.05, AA=African American, Cauc=Caucasian.

^a^ Scored on 1–5 scale where 1=graduated high school, 2 = 2 year college/vocational school, 3=graduated 4 year college, 4=some graduate/professional school, 5=completed graduate/professional school.

### Problematic substance use

Youths and their parents completed the Substance Use Domain of the Drug Use Screening Inventory- Revised (42) at baseline and follow-up. Follow-up assessments were conducted every six months after the baseline fMRI scan for up to 5 years following the baseline fMRI scan. Although follow-up was repeated, youths contributed a single outcome (yes or no) to indicate whether they had initiated problematic substance use at any point during the follow-up period (all participants were substance-naïve at baseline). The DUSI-R covered 25 substance classes, assessing monthly use frequency and endorsement of substance-specific problems. Additionally, youths also provided samples for a urine drug screen and alcohol breathalyzer (Uritox, LLC, Toledo, OH). Urine drug screens were coded dichotomously, with conservative coding applied for discrepancies (i.e., either positive urine drug screen results or self-reported use were coded as substance use), ensuring capture of any substance use.

Problematic substance use was defined as meeting ≥1 of the following criteria: frequent use of a substance (≥10 occasions/months), ≥2 DSM-5 consequences, unsafe use (legal problems, injury), or use of a potentially lethal substance (inhalants, cocaine, opioids). Youths not meeting these criteria were classified as youth without problematic substance use.

### Balloon analogue risk task

**Figure 1:**
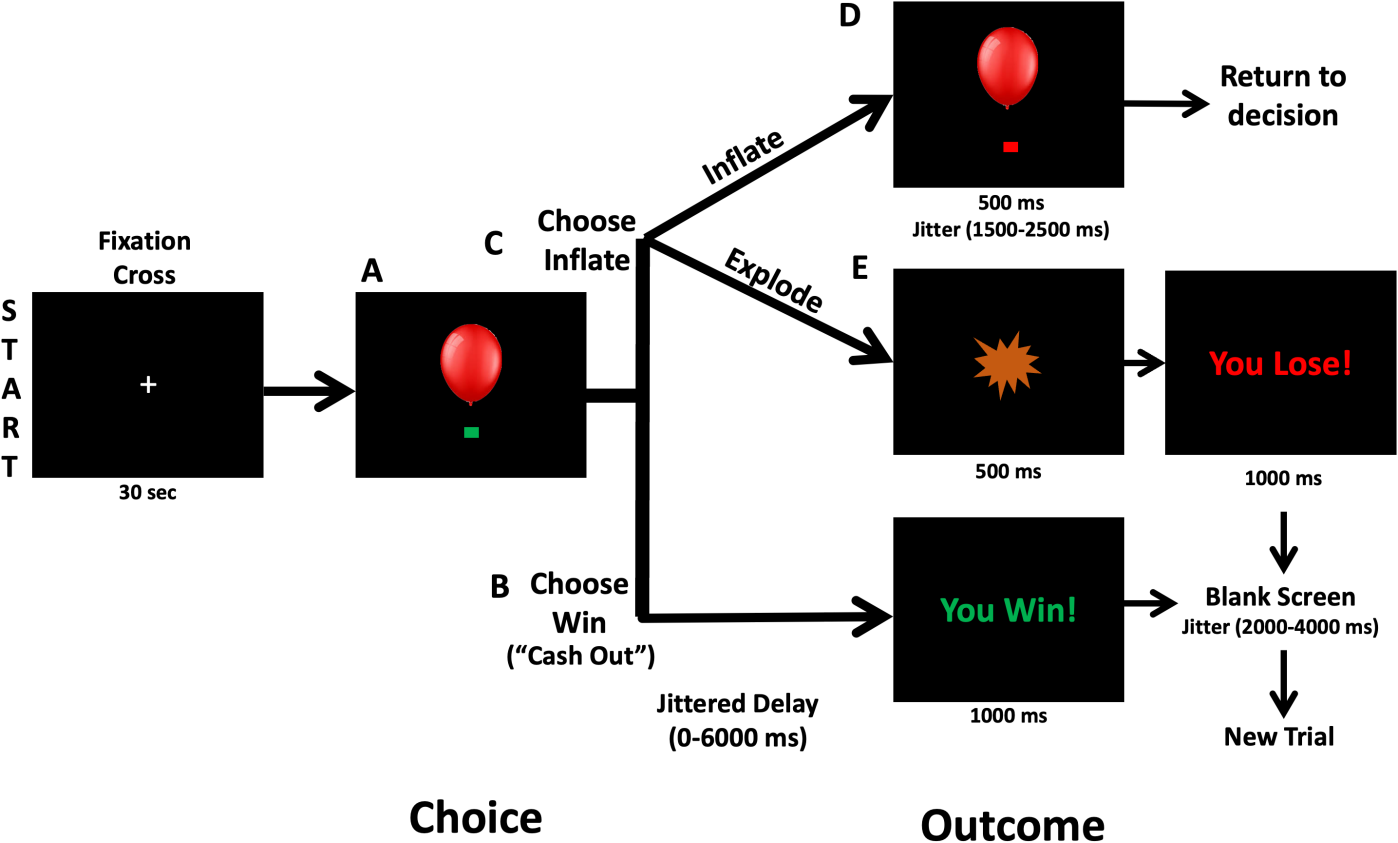
At the beginning of each Balloon Analogue Risk Task (BART) trial, a balloon and a green decision cue are shown on the screen. **(A)** Participants decide to inflate the balloon or cash out and take the accumulated wager via a button press. **(B)** Following Choose Win decisions, there is a 0–6000 ms jitter followed by a screen that says “You Win!” for 1000 ms. Then a 2000–4000 ms fixation is shown prior to starting a new balloon trial. **(C)** Following a decision to inflate the balloon (Choose Inflate), the balloon can either inflate or explode. **(D)** If the balloon inflates, an inflated balloon is shown for 1500–2500 ms before returning to the decision period and allowing another choice. **(E)** If the balloon explodes, participants are shown an explosion for 1500 ms and then a fixation cross for 2000–4000 ms before proceeding to the next balloon.

A version of the BART modified for use in MRI (35, 43) was administered via Eprime software (Psychology Software Tools, Pittsburgh, PA) to measure brain activation during risky decision-making. This task has been used in our prior work (33) and is fully described in **Figure 1** and the **Supplementary Material**.

### MRI data acquisition and preprocessing

For MRI parameters and preprocessing procedure, see **Supplementary Material**.

### MRI data first-level GLMs

First-level GLM analyses were implemented in AFNI (44). Choice events were modeled as the time point at which a participant presses the button to either inflate the balloon (Choose Inflate) or stop inflating and win the money (Choose Win). Outcome events were aligned to the time point when the balloon was successfully inflated (Outcome Inflate) or exploded (Outcome Explode), or when the individual received money after choosing to stop inflating the balloon (Outcome Win).

First, the three runs were concatenated prior to first-level modeling. Six head motion parameters and their derivatives, as well as a scanner drift term were modeled. Regressors were created by convolving the train of stimulus events with a double-gamma hemodynamic response function with a first-order temporal derivative to create a model Blood Oxygenation Level Dependent (BOLD) response time series for each condition. Five task-related regressors included: two choices (choose inflate, choose win), three outcomes (outcome win, outcome inflate, outcome explode); and a nuisance regressor (choice trials with reaction times>5000ms). This procedure generated β-coefficients/*t*-statistics for each voxel and regressor.

### Multiple comparison correction

All clusters were clusterwise corrected to *p* <.05 using an initial *p* <.001 and an extant threshold of *k* = 30 contiguous voxels. For further details regarding the multiple comparison correction procedure, see **Supplementary Material**.

### Data analysis

#### Demographics, VTE, and diagnostic data

We investigated differences in demographic characteristics (age, verbal IQ, performance IQ, Tanner Stage) via a series of t-tests with age, verbal IQ, performance IQ, and Tanner Stage as the dependent variables, respectively. The differences in child gender, race, and ethnicity between the groups were assessed via a series of chi-squared tests.

We also conducted a series of t-tests UCLA, SAVE-IV, SAVE-TV, and SAVE-PA scores as the dependent variables to check for group differences on VTE measures. We also repeated our factor extraction procedure with only the participants included in the sample to ensure that our latent variable behaved similarly in our fMRI sample versus our entire sample.

We conducted a series of chi-squared tests to test for differences in presence of Conduct Disorder, Oppositional Defiant Disorder, unspecified Disruptive Behavior Disorder, history of trauma-related or anxiety disorders, or problematic substance use at follow up.

Family History of SUD was included in the model to covary for family history effects, since the main study was aimed at investigating the effects of family history of SUD on risky decision-making, and VTE was associated with family history status (see **Table 1**).

#### Motion

We investigated group differences in head motion during fMRI via a Multivariate ANOVA with follow up t-tests. The dependent variables in the Multivariate ANOVA were number of censored volumes, average motion per volume, maximum displacement, and number of outliers.

#### BOLD Response fMRI data

We investigated group differences in BOLD responses via an ANOVA using AFNI’s 3dMVM (45). The main and interactive effects on BOLD responses during the BART task were examined using a 2 (VTE: Low, High) x 2 (Family History of SUD: No, Yes) x 2 (Choice: Inflate, Win) ANOVA. VTE and Family History were modeled as between-subjects factors while choice was modeled as a within-subjects factor. The contrasts of interest were represented within the overall ANOVA by the 2 (VTE: Low, High) x 2 (Choice: Inflate, Win) main and interaction effects. Family History of SUD was included in the model as a categorical predictor variable to account for the variance explained by family history, since the main study was aimed at investigating the effects of family history of SUD on risky decision-making, and VTE was associated with family history status (see **Table 1**).

*Post-hoc* analyses were conducted on the mean percent signal change extracted from all significant voxels within each functional ROI generated by AFNI to examine significant main effects and interactions with planned *post-hoc* testing within SPSS 28.0.

#### Problematic substance use outcomes

We investigated the relationship between BOLD response during the BART task and problematic substance use outcomes was tested using logistic regression. Our dependent variable was the binary outcome of problematic substance use, while the predictors were percent signal changes from all significant voxels within each functional ROI.

For this analysis, we also included age of onset of problematic substance use (or age of last report for youth without problematic substance use), given variable intervals between baseline fMRI visit and last follow up (as this is an ongoing longitudinal study).

Model performance was optimized for area under the curve (AUC). Class probabilities were extracted and thresholds for classifying individuals with or without problematic substance use were selected using Youden’s j and these thresholds were applied to classify participants.

## Results

### Demographics, VTE, and diagnostic data

There were no significant differences between the groups on age, verbal IQ, performance IQ, or Tanner Stage (*t*s<0.84, *p*s>.40). There were no significant differences in gender, race, or ethnicities across the groups (*χ*^2^s<2.38, *p*s>.12). See **Table 1** for details.

Individuals in the high VTE groups had greater scores on the UCLA, SAVE-IV, SAVE-TV, and SAVE-PA relative to individuals in the low VTE groups (*t*s>4.55, *p*s<.001). Additionally there was a correlation of *r* = 0.998 between factor scores extracted from the sample used in the fMRI analysis versus factor scores extracted from the entire sample.

There were no significant differences in CD, ODD, or other DBD diagnoses across the cells (*χ*^2^s<0.75, *p*s>.39). See **Table 1** for details.

BART Behavioral Data. See **Table 2** for analyses of behavioral data on the BART task.

**Table 2:** BART behavioral patterns by group.

	Lo VTE/FH-		Hi VTE/FH-		Lo VTE/FH+		Hi VTE/FH+		F-Statistic	Comparisons
	M	SD	M	SD	M	SD	M	SD		
Choices										
Number of Inflate Choices	264.6	34.7	270.6	27.07	262.0	37.01	271.4	28.27	0.34 1.09 0.08	
Number of Win Choices	39.1	11.97	37.7	10.98	39.8	11.93	36.6	11.92	1.11 4.38* 0.77	Lo VTE>Hi VTE
Reaction time (ms)										
Choose Inflate	1056.2	537.50	1016.4	392.22	1089.8	696.08	1045.0	329.69	0.75 0.19 0.08	
Choose Win	880.2	377.37	926.9	460.4	1170.6	889.13	899.4	417.55	1.76 0.00 0.24	

*indicates significant differences at *p* <.05, M = mean; SD = standard deviation.

### Motion

There were no significant multivariate effects of VTE on any motion parameters (*t*s<1.32, *p*s>.19).

### BOLD response data

Main effect of VTE: There was a significant main effect of VTE on BOLD response within right anterior insula. Individuals with high VTE showed more negative BOLD response within this brain region relative to individuals with low VTE. See **Table 3** for details.

**Table 3:** Brain regions demonstrating significant VTE and VTE-by-Choice effects.

Coordinates of peak activation^b^								
Region^a^	Hemisphere	BA	x	y	z	*F*	Partial η^2^	Voxels
Main effect of VTE								
AIC	R	13	40	6	3	11.73	0.133	44
VTE-by-Choice								
AIC	R	47	42	3	0	22.01	0.156	66
AIC/IFG	L	13	-28	26	-10	23.73	0.166	41
Caudate	L	–	-10	8	3	17.44	0.128	38

^a^ According to the Eckhoff-Zilles Macro Labels Atlas.

^b^ Based on the MNI standard brain template, VTE=Violence and Threat Exposure; BA= Brodmann’s Area; AIC=Anterior Insular Cortex; IFG=Inferior Frontal Gyrus; R=Right; L=Left.

**Figure 2:**
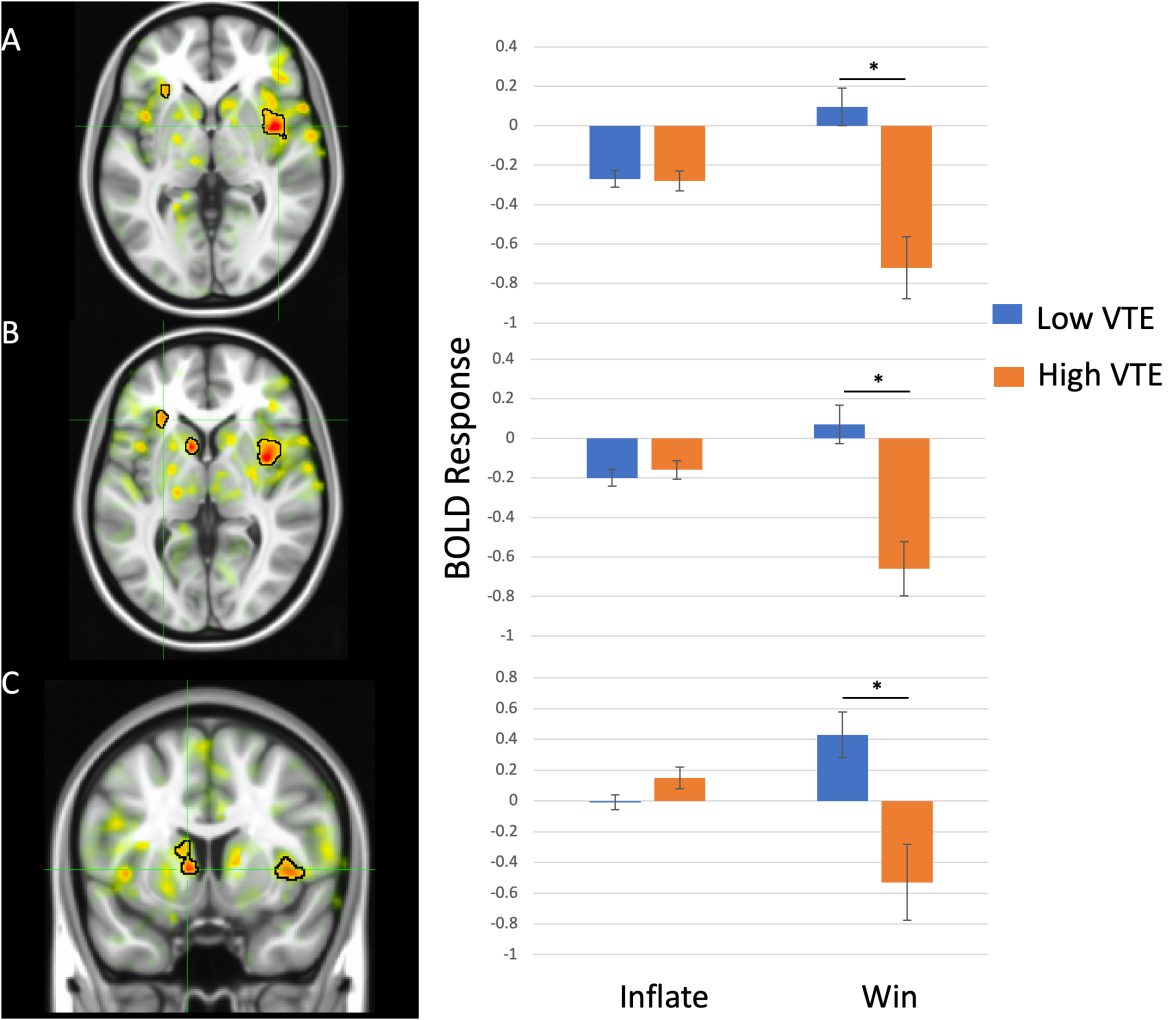
Violence and Trauma Exposure (VTE) group-by-Choice interaction effects within **(A)** right anterior insula **(B)** left anterior insula/inferior frontal gyrus and **(C)** left caudate. In all brain regions individuals with high VTE showed reduced BOLD response when choosing to win compared to choosing to inflate the balloon. * indicates p<.05.

VTE-by-Choice interaction: There was a significant VTE-by-Choice interaction effect on BOLD response within bilateral anterior insula, left inferior frontal gyrus, and left caudate nucleus. Within all three brain regions, individuals with high VTE showed reduced BOLD response when choosing to stop inflating the balloon (i.e., stop taking a risk) relative to individuals with low VTE (*t*s<-3.49, *p*s<.001), but no differences when choosing to inflate the balloon (i.e., proceed with taking a risk; *t*s<-1.91, *p*s>.05). See **Figure 2**, **Table 3** for details.

There were no main effects of family history of SUD or family history-by-choice interaction effects that survived multiple comparison correction.

### Prediction of problematic substance use

Our logistic regression model showed fair predictive performance (AUC = 0.74, Accuracy=0.694). BOLD response within right AIC/IFG during choosing to inflate the balloon was significantly associated with problematic substance use at follow up (B = 2.05, Wald z=5.63, *p* <.02). Additionally, age at first use was associated with problematic substance use (B = 0.41, Wald z=4.64, *p* <.05). No other variables were significantly associated with problematic substance use at follow up.

### Potential Confounds

As mentioned, we included family history of SUD as a categorical covariate of no interest in our main model since i) the main study is aimed at examining family history effects and ii) family history is associated with VTE in our sample.

Additionally, there were a number of potential confounds in our sample, including impulsivity and demographic variables, which are examined in our supplemental analyses. We also examined different ways in which to use VTE scores as predictors in our model, which is also examined in our supplemental analyses. None of these analyses substantially changed our results. See **Supplementary Material** for further information.

## Discussion

The aim of this study was to examine the effect of violence and threat exposure (VTE) on neural activity during risky decision-making in children with co-morbid ADHD and disruptive behavior disorders. Our hypothesis was that VTE would be associated with reduced activity within lateral frontal and striatal networks when engaging in risky decision-making. Our findings revealed that individuals with higher levels of VTE exhibited reduced activity within anterior insular cortex (aIC), inferior frontal gyrus (IFG), and caudate nucleus when deciding to not proceed with risk taking (i.e., stop inflating the balloon), in comparison to individuals with lower VTE while making choices. Additionally, activity within aIC was associated with problematic substance use at follow up.

Our prediction was that individuals with high levels of VTE would exhibit reduced activation in lateral frontal and striatal networks when making risky decisions compared to individuals with low VTE. The results of our study partially support this hypothesis, as individuals with high VTE displayed reduced BOLD response in right aIC, inferior frontal gyrus, and caudate when making the decision to stop inflating the balloon, in comparison to individuals with low VTE. The aIC is a crucial node of the salience network (46), encodes arousal to stimuli during decision-making tasks (47), and shows connectivity with the striatum that mediates action selection during decision-making (48). Recent research has shown that maltreatment, and specifically neglect, is associated with abnormalities in aIC activation during a lottery choice task across time (49). Moreover, we found that individuals with high levels of VTE show similar differences within caudate nucleus. Individuals with a history of institutional deprivation early in life show impairments neural representations of differences in reward magnitude (50) and reward response (10) within caudate nucleus. Furthermore, previous behavioral work using the BART has indicated behavioral impairments in children with VTE (51, 52). Another possibility is that these differences reflect alterations in safety signaling in individuals with high VTE. Prior work has shown that children with histories of maltreatment show reduced modulation by expected value within a cluster of lateral orbitofrontal cortex (adjacent to aIC and inferior frontal gyrus) during a passive avoidance task (16). However, it should be noted that these alterations may be context-dependent on VTE specifically and/or that individuals with PTSD or other stressor-related conditions may show different alterations (4, 53). Future work is necessary to disentangle these possibilities. Building upon prior work, our study expands the understanding of the effects of VTE by demonstrating that children with high levels of VTE exhibit reduced activation in the anterior insula when deciding to bank the money during the BART.

We also found that brain activity associated with VTE was predictive of problematic substance use at follow up in youths with disruptive behavior disorders. Specifically, increased activity within right aIC when choosing to inflate the balloon predicted problematic substance use at follow-up. Prior work examining the neurobiology mediating the relationship between ELA and substance use has found that alterations in resting-state connectivity within salience network plays a role in the development of substance use in young people who have experienced ELA (22, 31). Our data extend these findings by showing that alterations in activity within anterior insula during risky decision-making may play a role in the development of problematic substance use in youths who have experienced VTE. However, while alterations when choosing to stop inflating the balloon was associated with VTE, alterations when choosing to continue inflating the balloon was not associated with problematic substance use. These data indicate that while VTE may be associated with alterations within salience network, the nature of VTE’s relationship with problematic substance use outcomes may be more complex than can be captured within the current study.

The current study has several limitations. First, in this particular case, VTE was coded as a categorical variable, rather than a continuous variable. While this decision to categorize a continuous variable enhances the reliability of our fMRI analyses (54), transforming a continuous variable into a dichotomous variable reduces the power of our analyses. We conducted additional analyses using the continuous scores of the VTE latent variable as a predictor, as outlined in the **Supplementary Material**. Related, it could be argued that the selection of using a mean split to dichotomize VTE is arbitrary; therefore, we have re-run our analysis using a median split (see **Supplementary Material**). Our results were not substantially altered when using median rather than a mean split, indicating that our results are not a function of the specific cutoff we used to define higher versus lower VTE. Further, additional work is needed to explore the extent to which brain alterations related to other dimensions of adversity (deprivation, neglect, household dysfunction, poverty-related stress, etc.) may play a role in increasing risk for substance use. Furthermore, there were significant main effects of VTE on several factors underlying impulsivity (i.e., positive urgency, negative urgency, and sensation seeking). These findings indicate that the observed effects could be attributed to differences in impulsivity levels between the groups. To address this concern, we re-ran our analyses covarying for UPPS scores. These supplementary analyses largely replicated our main findings, as detailed in the **Supplementary Material**, thus mitigating this concern. Additionally, the current study focused on children with co-morbid ADHD and disruptive behavior disorders, as these children have a high likelihood of experiencing VTE (29, 55). While clinically relevant, this may limit generalizability of the current findings to typically developing populations. Also, we did not directly examine the effects of SES, as income data were available for only 75 participants. Future investigations should examine the role of SES in the associations between VTE and altered brain functioning. It should also be noted that there were no behavioral differences between groups on the BART task. This is in line with prior work on reinforcement learning in VTE (10). One possibility is that current decision-making and reinforcement paradigms are not sensitive enough to pick up behavioral effects associated with VTE. A second possibility is that individuals with high levels of VTE are compensating in some form for neural level issues in the encoding of risk. Also, the assessment of VTE experiences relied solely on self-report measures, without corroborating reports from local Child Protective Services. It is important to acknowledge that some individuals who have experienced VTE may not have disclosed or reported their experiences. Another limitation is that our definition of externalizing disorders (ADHD and one or more DBD diagnoses) does not allow us to disentangle ADHD vs DBD comorbidity effects. Future work should consider whether ADHD or DBD diagnoses moderate the effect of ELA on brain functioning and substance use risk. Finally, caution is warranted regarding our analyses predicting problematic substance use. Since we used ROIs defined from the BART task in the same sample, this particular finding should be regarded as exploratory and hypothesis-generating, rather than confirmatory. Future work using *a priori* ROIs in a separate sample should be used to confirm this finding.

In summary, our findings provide evidence of neural alterations during risky decision-making in children with greater VTE. Specifically, we observed altered activation patterns within the anterior insula regions when children with VTE made decisions during the BART and that these activation patterns predict problematic substance use. These data may suggest that children with VTE have alterations in generating neural signals that help differentiate between safer and riskier options, resulting in impairments in goal-directed behaviors and potentially predisposing them to problematic substance use in adolescence. The results contribute to the expanding body of literature that investigates decision-making impairments in children with VTE and their predisposition to problematic substance use.

## Data Availability

The original contributions presented in the study are included in the article/**Supplementary Material**, further inquiries can be directed to the corresponding author/s.
