# Supplementary figures and images for: Violence and threat exposure is associated with frontostriatal alterations during risky decision-making in children with co-morbid ADHD and disruptive behavior disorders
Source: Front Psychiatry. 2026 May 8;17:1799471. doi: 10.3389/fpsyt.2026.1799471 (PMC13194102; doi:10.3389/fpsyt.2026.1799471)

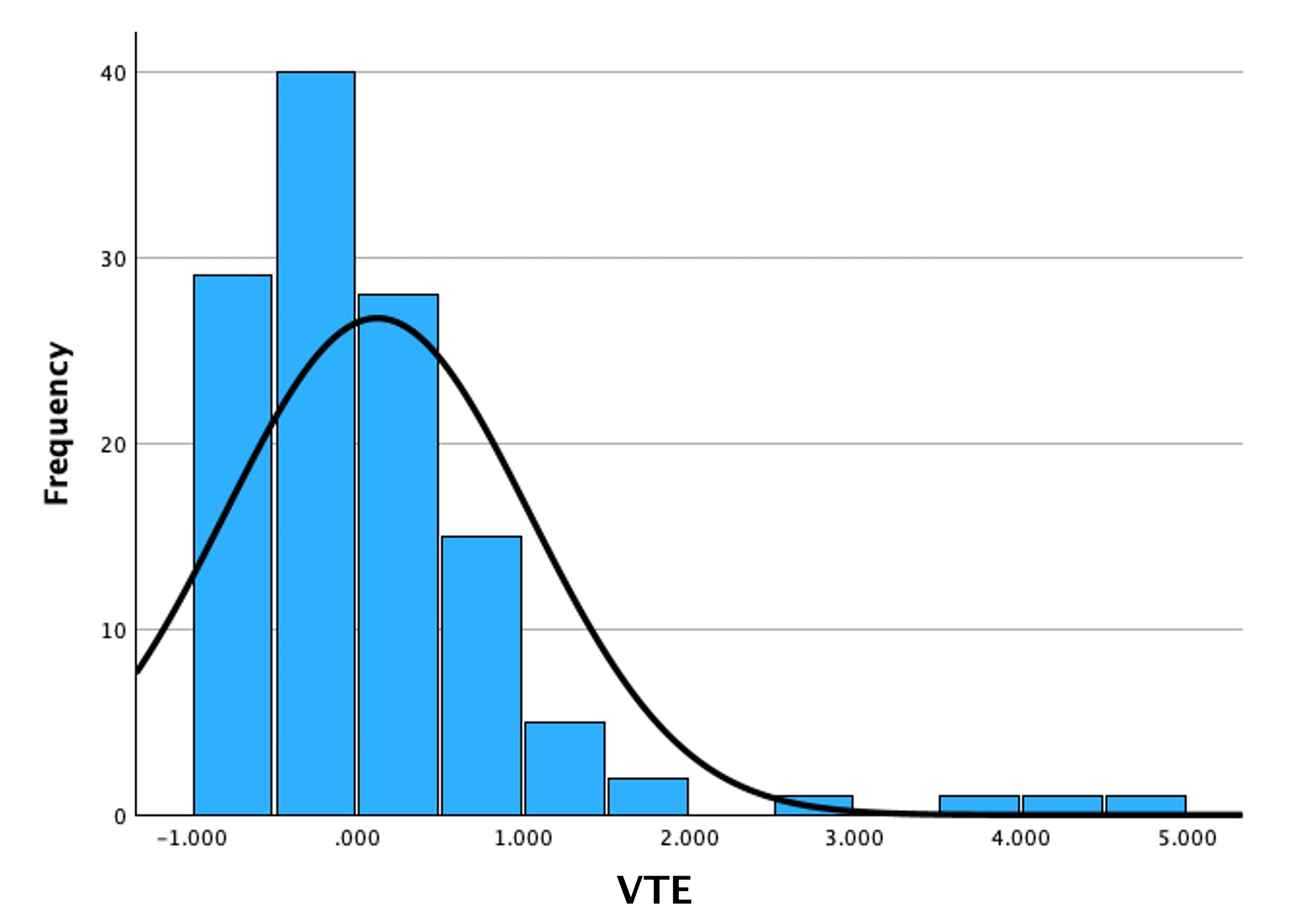

Supplement: Supplementary file 1 [file Image1.tiff]
