# Supplementary material for: Violence and threat exposure is associated with frontostriatal alterations during risky decision-making in children with co-morbid ADHD and disruptive behavior disorders
Source: Front Psychiatry. 2026 May 8;17:1799471. doi: 10.3389/fpsyt.2026.1799471 (PMC13194102; doi:10.3389/fpsyt.2026.1799471)
Supplement: Supplementary file 2 [file Table1.docx]

**Supplement**

**Supplemental Methods**

**Exclusion Criteria**

Exclusion criteria for the study were: meeting DSM-5 criteria for a current (within the last month) mood disorder and/or individuals with any lifetime history of psychotic symptoms, bipolar disorder, autism spectrum disorder, substance use, neurological problems, debilitating medical conditions, estimated total IQ<80, or MRI contraindications (e.g., claustrophobia, metal implants).

**VTE Latent Variable Analysis**

A histogram depicting the distribution of z-scores of children used in the fMRI analysis is depicted in Figure S1, showing that the distribution of these scores was continuous (rather than bimodal). The factor loadings of each of the subscales on our VTE latent variable were as follows: SAVE-Traumatic Violence=0.80, SAVE-Physical/Verbal Abuse=0.77, SAVE-Indirect Violence=0.71, UCLA=0.61.

**Balloon Analogue Risk Task**

In the Balloon Analogue Risk Task (BART) task, participants must choose whether to 1) inflate a balloon to risk monetary reward that increases with each successive balloon inflation or 2) to win and bank the amount of money they have already made and start a new balloon. Participants saw a simulated balloon and pressed one of two buttons to either inflate the balloon (Choose Inflate), risking cash rewards that increase with the size of the balloon, or stop inflating and bank the accumulated money (Choose Win) and start a new balloon. If selecting “Choose Inflate”, there is a 0-6000ms jittered delay, after which the balloon image either inflates and increases the accumulated rewards (Outcome Inflate; displayed for 500ms + 1500ms-2500ms jitter) or explodes and loses accumulated money (Outcome Explode; displays a 500ms explosion+1000ms screen that reads “You Lose!”). Except for the first inflation, explosions can occur at any balloon size, but the risk of explosion increases as the balloon size increases. Following an explosion, a new balloon is started. If participants choose to win the money, accumulated cash rewards are banked, and a new balloon is presented. Over three eight-minute fMRI runs, participants completed as many balloons as possible. A maximum of 12 inflations were possible per balloon. Before the administration of the BART, participants practiced the task outside the scanner on a computer and understood that they would be paid with cash after the scan based on their performance. Last, we measured reaction time separately for the time duration that participants took to make a choice and push the button for Choose Inflate or Choose Win.

**MRI Data Acquisition**

Scans were conducted using a 3-Tesla Siemens Prisma MRI scanner with a 32-channel head coil. Co-registration and normalization of functional image volumes to MNI space were completed using a high-resolution 3D magnetization prepared rapid gradient echo (MPRAGE; 160 sagittal slices; 1.05×1.05×1.2 mm^3^ voxels) scan. Blood Oxygenation Level Dependent (BOLD) Functional MRI was performed using a T2*-weighted multiband (MB) gradient echo-planar imaging (EPI) sequence (Xu et al., 2013) (Repetition/Echo Time (TR/TE) 1200/29ms; flip angle 65^o^; field-of-view 220x220mm^2^; matrix 88x88, MB slice acceleration factor 3; 54 axial slices; voxel size 2.5×2.5×2.5mm^3^; 400 BOLD volumes). A pair of 16-second phase-reversed spin echo EPI scans (TR/TE 1560/48ms; 3 A-to-P and 3 P-to-A volumes) was acquired prior to functional imaging at the same locations and voxel size as the BOLD EPI acquisition.

**MRI Data Preprocessing** First, all EPI scans underwent a correction for susceptibility-induced distortions and magnetic field inhomogeneities using FMRIB Software library (FSL version 6.0.1) programs *topup/applytopup (*utilizing phase-reversed spin echo field mapping scans) (Woolrich et al., 2009). Then, the following preprocessing steps were completed using Analysis of Functional NeuroImages (Cox, 1996): i) the first five volumes collected prior to magnetization equilibrium were discarded; ii) each participant’s anatomical (T1-weighted) scan was registered to the MNI152 standard space template; iii) each participant’s EPI data were registered to their MNI anatomical scan; iv) functional images were motion corrected to the initial volume of the first functional run; v) functional images were spatially smoothed with an isotropic 6mm full-width at half-maximum Gaussian kernel; vi) EPI data then underwent time-series normalization to a T1-weighted image, and the voxel values were multiplied by 100 for each voxel. Finally, using FSL, we applied an unsupervised ICA-AROMA classifier (Pruim et al., 2015) to ensure robust data cleaning by using Independent Components Analysis to identify components in each individual’s dataset that were associated with noise and to discard these components from further analysis.

**Multiple Comparison Correction**

All clusters were clusterwise corrected to *p*<.05. Multiple comparison correction was performed using a spatial clustering operation in AFNI’s 3dClustSim utilizing the autocorrelation function (-acf) with 10,000 Monte Carlo simulations for the whole-brain analysis. Spatial autocorrelation was estimated using the residuals from the individual-level GLM analyses. The initial voxelwise threshold was set at *p*=.001. This procedure yielded an extant threshold of *k*=30 contiguous voxels for the whole-brain analysis (NN1/facewise neighbor clustering) (Cox, Chen, Glen, Reynolds, & Taylor, 2017a, 2017b).

**Supplemental Results**

**UPPS Scores**

*Methods*

We found significant differences in three full UPPS subscales (negative urgency, positive urgency, and sensation seeking) in the Hi VTE group vs. the Lo VTE group (see Table 1 in the main text). To address the possibility that our findings may have been confounded by elevated impulsivity in the Hi VTE group, we repeated our analyses co-varying for UPPS average score across all scales (since 3/5 scales were significantly associated with VTE group). As such, we conducted a 2 (FH-SUD: Negative vs. Positive) ×2 (VTE: Low vs. High) ×2 (Choice: Inflate, Win) ANCOVA with UPPS scores as a covariate on the BOLD responses.

*Results*

*Main Effect of VTE*

We found a significant main effect of VTE within AIC and IFG controlling for UPPS scores. Individuals in the high VTE group showed more negative BOLD response relative to individuals with low VTE. See Table S1 for further details.

*VTE-by-Choice Interaction*

We found a significant VTE-by-Choice interaction effect within AIC, IFG, caudate, insula, putamen, and dlPFC controlling for UPPS scores. Within all brain regions, individuals with high VTE showed reduced BOLD response when choosing to stop inflating the balloon relative to individuals with low VTE. See Table S1 for further details.

**Child Gender**

*Methods*

It is also possible that our findings may be related to demographic variables, such as gender or IQ. To address this possibility, we repeated our analyses with child gender as a categorical covariate. As such, we conducted a 2 (FH-SUD: Negative vs. Positive) ×2 (VTE: Low vs. High) ×2 (Child Gender: male, female) ×2 (Choice: Inflate, Win) ANOVA on the BOLD responses.

*Results*

*Main Effect of VTE*

We found a significant main effect of VTE within AIC controlling for Gender. Individuals in the high VTE group showed more negative BOLD response relative to individuals with low VTE. See Table S2 for further details.

*VTE-by-Choice Interaction*

We found a significant VTE-by-Choice interaction effect within AIC, IFG, and caudate controlling for Gender. Within all brain regions, individuals with high VTE showed reduced BOLD response when choosing to stop inflating the balloon relative to individuals with low VTE. See Table S2 for further details.

**Verbal IQ**

*Methods*

It is also possible that our findings may be related to demographic variables, such as gender or IQ. To address this possibility, we repeated our analyses with verbal IQ as a quantitative covariate. As such, we conducted a 2 (FH-SUD: Negative vs. Positive) x2 (VTE: Low vs. High) x2 (Choice: Inflate, Win) ANCOVA with verbal IQ as a covariate on the BOLD responses.

*Results*

*Main Effect of VTE*

We found a significant main effect of VTE within AIC, IFG, and postcentral gyrus controlling for verbal IQ. Individuals in the high VTE group showed more negative BOLD response relative to individuals with low VTE. See Table S3 for further details.

*VTE-by-Choice Interaction*

We found a significant VTE-by-Choice interaction effect within AIC, IFG, and caudate controlling for verbal IQ. Within all brain regions, individuals with high VTE showed reduced BOLD response when choosing to stop inflating the balloon relative to individuals with low VTE. See Table S3 for further details.

**VTE Latent Variable as a Continuous Variable**

*Methods*

Also, since we dichotomized the VTE latent variable, we present a supplemental analysis with VTE as a continuous variable rather than a categorical variable. As such, we conducted a 2 (FH-SUD: Negative vs. Positive) ×2 (Choice: Inflate, Win) ANCOVA with the z-scored VTE latent variable as a covariate on the BOLD responses.

*Results*

*Main Effect of VTE*

We found a significant main effect of VTE within inferior occipital gyrus. There was an inverse correlation between VTE and unmodulated BOLD response. See Table S4 for further details.

*VTE-by-Choice Interaction*

We found a significant VTE-by-Choice interaction effect within IFG. Within all brain regions, there was an inverse correlation between VTE and BOLD response when choosing to stop inflating the balloon. See Table S4 for further details.

**Dichotomizing VTE Group based on Median Split rather than Mean Split**

*Methods*

Also, since we dichotomized the VTE latent variable based on a mean split, we performed a supplemental analysis dichotomizing VTE based on a median split rather than a mean split to mitigate the concern that our results are an artifact of our selected cut point. As such, we conducted a 2 (FH-SUD: Negative vs. Positive) ×2 (VTE: Low vs. High) ×2 (Choice: Inflate, Win) ANOVA on the BOLD responses.

*Results*

*Main effect of VTE*

There was a significant main effect of VTE on BOLD response within right anterior insula. Individuals with high VTE showed more negative BOLD response within this brain region relative to individuals with low VTE. See Table S5 for details.

*VTE-by-Choice Interaction*

There was a significant VTE-by-Choice interaction effect on BOLD response within anterior insula, inferior frontal gyrus, precentral gyrus, middle cingulate cortex, superior temporal gyrus, and supplementary motor area. Within all brain regions, individuals with high VTE showed reduced BOLD response when choosing to stop inflating the balloon relative to individuals with low VTE. See Table S5 for details.

| Table S1. Brain regions demonstrating significant VTE and VTE-by-Choice Effects Covarying for Average UPPS Score | | | | | | | | |
| --- | --- | --- | --- | --- | --- | --- | --- | --- |
| Coordinates of Peak Activation^b^ | | | | | | | | |
| Region^a^ | Hemisphere | BA | x | y | z | *F* | Partial η^2^ | Voxels |
| Main Effect of VTE | | | | | | | | |
| AIC | R | 13 | 40 | 6 | 3 | 18.45 | 0.135 | 50 |
| IFG | R | 10/46 | 37 | 36 | 8 | 19.19 | 0.131 | 39 |
| VTE-by-Choice | | | | | | | | |
| AIC/IFG | R | 10/47 | 45 | 43 | -2 | 27.16 | 0.187 | 114 |
| Caudate | R | - | 12 | 6 | 5 | 25.18 | 0.176 | 106 |
| Insula | R | 13 | 37 | 1 | 0 | 23.84 | 0.168 | 83 |
| Caudate | L | - | -10 | 11 | 3 | 21.48 | 0.154 | 82 |
| dlPFC | R | 9/45/46 | 50 | 18 | 23 | 22.04 | 0.157 | 70 |
| Putamen | L | - | -20 | 8 | -5 | 21.12 | 0.152 | 41 |

Note: ^a^ According to the Eckhoff-Zilles Macro Labels Atlas, ^b^ Based on the MNI standard brain template,
BA= Brodmann’s Area

| Table S2. Brain regions demonstrating significant VTE and VTE-by-Choice Effects Covarying for Gender | | | | | | | | |
| --- | --- | --- | --- | --- | --- | --- | --- | --- |
| Coordinates of Peak Activation^b^ | | | | | | | | |
| Region^a^ | Hemisphere | BA | x | y | z | *F* | Partial η^2^ | Voxels |
| Main Effect of VTE | | | | | | | | |
| AIC | R | 13 | 42 | 3 | 0 | 19.84 | 0.144 | 39 |
| VTE-by-Choice | | | | | | | | |
| AIC | R | 13 | 42 | 3 | 0 | 22.03 | 0.157 | 59 |
| IFG | R | 46 | 40 | 36 | 8 | 25.43 | 0.177 | 43 |
| Caudate | L | - | -10 | 11 | 3 | 16.91 | 0.125 | 38 |

Note: ^a^ According to the Eckhoff-Zilles Macro Labels Atlas, ^b^ Based on the MNI standard brain template,
BA= Brodmann’s Area

| Table S3. Brain regions demonstrating significant VTE and VTE-by-Choice Effects Covarying for Verbal IQ | | | | | | | | |
| --- | --- | --- | --- | --- | --- | --- | --- | --- |
| Coordinates of Peak Activation^b^ | | | | | | | | |
| Region^a^ | Hemisphere | BA | x | y | z | *F* | Partial η^2^ | Voxels |
| Main Effect of VTE | | | | | | | | |
| AIC | R | 13 | 42 | 6 | 0 | 18.99 | 0.141 | 50 |
| IFG | R | 46 | 40 | 36 | 8 | 19.24 | 0.142 | 33 |
| Postcentral Gyrus | R | 43 | 50 | -9 | 18 | 14.34 | 0.110 | 32 |
| VTE-by-Choice | | | | | | | | |
| AIC | R | 13 | 42 | 3 | 0 | 21.91 | 0.159 | 77 |
| AID/IFG | L | 47 | -28 | 26 | -10 | 23.94 | 0.171 | 43 |
| Caudate | L | - | -10 | 8 | 3 | 17.31 | 0.130 | 42 |

Note: ^a^ According to the Eckhoff-Zilles Macro Labels Atlas, ^b^ Based on the MNI standard brain template,
BA= Brodmann’s Area

| Table S4. Brain regions demonstrating significant VTE and VTE-by-Choice Effects With VTE as a Continuous Covariate | | | | | | | | |
| --- | --- | --- | --- | --- | --- | --- | --- | --- |
| Coordinates of Peak Activation^b^ | | | | | | | | |
| Region^a^ | Hemisphere | BA | x | y | z | *F* | Partial η^2^ | Voxels |
| Main Effect of VTE | | | | | | | | |
| Inferior Occipital Gyrus | L | 37 | -40 | -69 | -5 | 20.47 | 0.147 | 45 |
| VTE-by-Choice | | | | | | | | |
| IFG | R | 44 | 60 | 13 | 10 | 20.62 | 0.148 | 53 |

Note: ^a^ According to the Eckhoff-Zilles Macro Labels Atlas, ^b^ Based on the MNI standard brain template,
BA= Brodmann’s Area

| Table S5. Brain regions demonstrating significant VTE and VTE-by-Choice Effects with VTE Dichotomized based on Median split | | | | | | | | |
| --- | --- | --- | --- | --- | --- | --- | --- | --- |
| Coordinates of Peak Activation^b^ | | | | | | | | |
| Region^a^ | Hemisphere | BA | x | y | z | *F* | Partial η^2^ | Voxels |
| Main Effect of VTE | | | | | | | | |
| AIC | R | 13 | 40 | 1 | 0 | 17.00 | 0.125 | 31 |
| VTE-by-Choice | | | | | | | | |
| AIC | R | 13 | 42 | 3 | 0 | 27.75 | 0.189 | 112 |
| IFG | L | 46 | -48 | 31 | 10 | 22.86 | 0.161 | 76 |
| IFG | R | 47 | 47 | 33 | -2 | 23.91 | 0.167 | 56 |
| Precentral Gyrus | R | 6 | 27 | -22 | 73 | 22.28 | 0.158 | 46 |
| Middle Cingulate Cortex | L | 31 | -8 | -14 | 48 | 18.70 | 0.136 | 39 |
| Superior Temporal Gyrus | R | 22 | 64 | -2 | -2 | 18.69 | 0.136 | 35 |
| Supplementary Motor Area | L | 6 | -3 | 11 | 63 | 18.52 | 0.135 | 35 |
| Precentral Gyrus | R | 44 | 55 | 1 | 23 | 19.32 | 0.140 | 32 |

Note: ^a^ According to the Eckhoff-Zilles Macro Labels Atlas, ^b^ Based on the MNI standard brain template,
BA= Brodmann’s Area

***UCLA Trauma History Self-Report***

The following nine items from the UCLA Trauma History Self-Report instrument were used. This was scored on a scale of 0-9, where a “Yes” on each item was coded as 1 and a “No” on each item was coded as a 0, and these values were summed.

***1. Sometimes people have scary or violent things that happen to them where someone could have been or was badly hurt or killed. Has anything like this ever happened to you?***

□Yes

□No

*Below is a list of other scary or violent things that can happen. For each question, check* ***“Yes”*** *if this has* ***happened to you***; *check* ***“No”*** *if this* ***did NOT happen to you***

2. Were you in a disaster, like an earthquake, wildfire, hurricane, tornado or flood?

□Yes

□No

3. Were you in a bad accident, like a serious car accident or fall?

□Yes

□No

4. Were you in a place where a war was going on around you?

□Yes

□No

5. Were you hit, punched, or kicked very hard at home?

(DOES NOT INCLUDE play fighting between brothers and sisters.)

□Yes

□No

6. Did you see a family member being hit, punched or kicked very hard at home?

(DOES NOT INCLUDE play fighting between brothers and sisters).

□Yes

□No

7. Were you beaten up, shot at, or threatened to be hurt badly in your school,

neighborhood or town?

□Yes

□No

8.Did you see someone who was beaten up, shot at or killed?

□Yes

□No

9. Did you see a dead body (do not include funerals)?

□Yes

□No

**Supplemental Figure Legend**

**Figure S1.** Distribution of the Early Life Stress (VTE) latent variable scores. Individuals with VTE scores <0 were categorized into the low VTE group while individuals with VTE scores >0 were categorized into the high VTE group.

**References**

Cox, R. W. (1996). AFNI: Software for analysis and visualization of functional magnetic resonance neuroimages. *Computers and Biomedical Research, 29*(3), 162-173. doi:10.1006/cbmr.1996.0014

Cox, R. W., Chen, G., Glen, D. R., Reynolds, R. C., & Taylor, P. A. (2017a). fMRI clustering and false-positive rates. *Proc Natl Acad Sci U S A, 114*(17), E3370-E3371. doi:10.1073/pnas.1614961114

Cox, R. W., Chen, G., Glen, D. R., Reynolds, R. C., & Taylor, P. A. (2017b). FMRI Clustering in AFNI: False-Positive Rates Redux. *Brain Connect, 7*(3), 152-171. doi:10.1089/brain.2016.0475

Pruim, R. H. R., Mennes, M., van Rooij, D., Llera, A., Buitelaar, J. K., & Beckmann, C. F. (2015). ICA-AROMA: A robust ICA-based strategy for removing motion artifacts from fMRI data. *Neuroimage, 112*, 267-277. doi:10.1016/j.neuroimage.2015.02.064

Woolrich, M. W., Jbabdi, S., Patenaude, B., Chappell, M., Makni, S., Behrens, T., . . . Smith, S. M. (2009). Bayesian analysis of neuroimaging data in FSL. *Neuroimage, 45*(1 Suppl), S173-186. doi:10.1016/j.neuroimage.2008.10.055

Xu, J., Moeller, S., Auerbach, E. J., Strupp, J., Smith, S. M., Feinberg, D. A., . . . Ugurbil, K. (2013). Evaluation of slice accelerations using multiband echo planar imaging at 3 T. *Neuroimage, 83*, 991-1001. doi:10.1016/j.neuroimage.2013.07.055
